# Measuring financial sector efficiency in China: A study based on an enhanced neoclassical production function

**DOI:** 10.1371/journal.pone.0319480

**Published:** 2025-04-10

**Authors:** Chuanzhen Zheng, Yuan Shan

**Affiliations:** 1 School of Economics and Management, Tongji University, Shanghai, China; 2 Faculty of Economics and Business Administration, Ghent University, Ghent, Belgium; Southwestern University of Finance and Economics, CHINA

## Abstract

This paper develops an approach for quantitatively assessing financial sector efficiency of a nation (hereinafter referred to as financial efficiency). Drawing upon the principles of general equilibrium theory, we establish a direct and strong association between the financial sector and the real sector by improving the neoclassical production function, indirectly unveiling insights into financial efficiency through the allocation structure of marginal capital output. We use a two-stage semiparametric estimation method to determine the structural parameters of the production function in the calculation of financial efficiency. The values of financial efficiency for China, spanning the years 1997–2018, exhibit a distinct pattern of initially increasing, followed by a decline, and subsequent resurgence, culminating in the highest recorded value in 2008. Prior to 2015, the financial efficiency closely mirrored the trajectories of total factor productivity (TFP) and GDP growth rate. However, post-2015, we observe a discernible deviation in the trend of financial efficiency from these two economic indicators. These results can be verified by external empirical facts and evidence.

## Introduction

Financial efficiency is defined as the relationship between inputs and outputs in the allocation of financial resources, specifically the efficiency of resource allocation between suppliers and demanders. Studies examining financial efficiency can be categorized into two distinct domains, contingent upon the specific subjects of analysis. The first category entails the assessment of financial efficiency within individual entities, such as enterprises or banks [1]. The second category revolves around the evaluation of financial sector efficiency within a nation or region [2]. This paper falls under the purview of the latter category. Financial efficiency emerges as a pivotal gauge of financial development maturity, with shifts in financial efficiency playing a critical role in discerning the degree of financial development and forecasting economic growth trajectories [3–5]. In particular, Jeanneney [6] find that financial development significantly supports China’s productivity growth by increasing its efficiency. Thus, there are close linkages between financial efficiency and economic growth and productivity growth.

How can financial efficiency be measured at the national level? It is essential to first define the concept of financial efficiency and establish measurement methods. In this paper, financial efficiency pertains to the effectiveness of capital allocation from the financial sector to the real sector, meaning that a country-level assessment of financial efficiency measures the efficiency of national capital allocation. We contend that, at the national level, financial efficiency should reflect the aggregate effect of the financial sector within the capital allocation system, as opposed to solely reflecting the efficiency of an organization in allocating its own capital. Therefore, selecting indicators such as the proportion of certain financial data to GDP to gauge national-level financial efficiency is subject to significant subjectivity and lacks a robust economic theoretical foundation. Moreover, it fails to elucidate economic mechanisms and economic significance. When capital exists in a monetary form, such as credit, it may not accurately reflect financial efficiency since capital can be embedded or idle within the financial system. To address these concerns, we adopt an indirect approach to measuring financial efficiency, which establishes a link between the financial sector and the real sector through capital flows. Our indirect method calculates the marginal output of capital and the average return on capital from the output and income sides. We express financial efficiency as the reciprocal of the ratio of the difference between these two measures to the marginal output of capital.

Many quantitative methods for assessing financial efficiency may fall short in utilizing feedback from the real sector [5,7]. This study establishes a strong association between capital allocation in the financial sector and the real sector when evaluating financial sector efficiency within a nation. In macroeconomic models, first-order derivatives for the representative firm indicate that the efficiency of capital allocation is maximized when the marginal output of capital equals the unit return on capital (price of capital) [8]. When the unit return on capital (referred to in our analysis as the average return on capital) is lower than the marginal output of capital, it signifies that a portion of the output generated by capital is captured by the financial sector. For example, China’s financial market structure exhibits a blend of oligopoly and monopolistic competition, allowing major commercial banks in such a market structure to accrue substantial surpluses [9,10]. If such profits were attained in a perfectly competitive market, it would be beneficial for the banks themselves, businesses, and the entire society. However, in the context of oligopoly and monopolistic competition market structures, the higher the proportion of output seized by the financial sector in relation to the marginal output of capital, the lower the financial efficiency of the sector.

The approach of this paper is mostly related to the works of semi-parametric two-step estimation methods [11] and simultaneously addressing input and productivity determination issues [12]. Semi-parametric two-stage estimation avoids strong assumptions about the distribution of the data and provides better handling of complex data. The simultaneous consideration of the input and productivity determination problem is an effective approach for addressing the endogeneity issue in TFP estimation, thereby enhancing the precision of financial efficiency estimates.

Possible innovations are as follows: (1) our approach establishes a direct and strong link between the financial and real sectors, thereby enhancing the precision of financial sector efficiency measures employed in financial efficiency studies. (2) our model integrates significant institutional changes and unexpected events that may affect TFP, thus accounting for shifts in productivity. These enhancements improve the accuracy of financial efficiency estimates. (3) we introduce novel adjustments to capital and analyze the effects of both physical and human capital accumulation on productivity. Further, we estimate the structural parameters of the production function, ensuring that our approach mitigates the impact of extraneous variables on the estimation of financial efficiency.

## Literature review

This article relates to at least three strands of literature. The first is research on the fitting of a country’s production function, which is a prerequisite for calculating marginal output of capital, one of the key variables in calculating financial efficiency. The central issue in production function research revolves around the estimation of structural parameters and the reduction or elimination of estimation errors, typically within the classical framework developed by Solow [13]. In response to the limitations of the Solow model, some scholars have enriched the literature on production functions by addressing endogeneity stem from measurement errors and omitted variables. Earlier studies have made significant strides in addressing measurement errors. For example, Young [14] demonstrates that human capital accumulation, as gauged by educational attainment, enhances effective labor and subsequently boosts output. Olley and Pakes [15] contend that variations in labor inputs significantly influence labor coefficients. Fernald (Unpublished) suggests that the significance of factor accumulation may have been undervalued in Hsieh’s [16] analysis. To address the problem of omitted variables, Levinsohn and Petrin [17] introduce a methodology utilizing intermediate inputs, thereby refining the measurement of factor inputs and productivity. Van Biesebroeck [18] overcomes the difficulty of unobservability of technology and productivity and finds that new technologies are more capital-biased and have higher capital-biased productivity growth.

Second, regarding estimation methods, many scholars contend that methodological enhancements have improved the accuracy of production function estimations. Olley and Pakes [15] introduce a pioneering two-step estimation method, which is further refined by Levinsohn and Petrin [17] and Ackerberg et al. [19]. Ackerberg et al. [11] conduct a comparative analysis, demonstrating that semi-parametric two-step estimation offers superior efficiency for estimation problems with uncertain parameter forms. Ackerberg et al. [19] argue that the assumptions and functional forms utilized in the OP (Olley and Pakes) method limit its capacity to accurately estimate labor coefficients. In response, Hu et al. [20] suggest an alternative methodology employing two mutually independent instrumental variables to capture and control unobserved factors that traditional models overlook. Kim et al. [12] find that the measurement errors of capital in both the OP and LP (Levinsohn and Petrin) methods can preclude the simultaneous determination of inputs and productivity. To resolve this issue, they develop consistent estimators for production function parameters.

Third, this paper also makes a valuable contribution to the growing body of literature on the macro-level financial efficiency. Zhang et al. [21] utilize the stochastic frontier model to evaluate financial efficiency at the county level in China. Hu et al. [5] define financial efficiency as the efficiency of financial resource allocation by financial institutions. Yuan et al. [2] measure financial efficiency at the provincial level in China by the ratio of gross capital formation to the total deposits of financial institutions. The methodology employed in this study measuring financial efficiency is based on a macro-financial sector perspective, which is significantly different from existing studies..

## Data and methodology

### Data

The data set used for this study covers the period from 1997 to 2019. Financial efficiency calculations, however, are restricted to the period ending in 2018 due to limitations in the availability of certain foundational data. To maintain data continuity and comparability, we have adjusted data points that required normalization, using 1997 as the base year for adjustment. The fundamental data necessary for the calculation of financial efficiency are provided in the following section.

### Labor and human capital

The data on the labor force are directly sourced from the annual *China Labor Statistics Yearbook* for each respective year. Human capital generally encompasses the accumulation of education and experience [22,23]. Since there is no readily available direct data for measuring the effect of experience (“learning by doing” [24]), the information available for human capital accumulation is based on the educational attainment of employed individuals, specifically their years of schooling [25–27]. This data, measured by schooling years (*s*) and its expected value E(*s*), has been published by the *National Institute of Statistics* since the end of the last century. The human capital stock is approximated by the logarithmic transformation *h =  ln(s)·l*, which is based on the decreasing marginal learning efficiency curve with the length of education [28,29].

**Fig 1:**
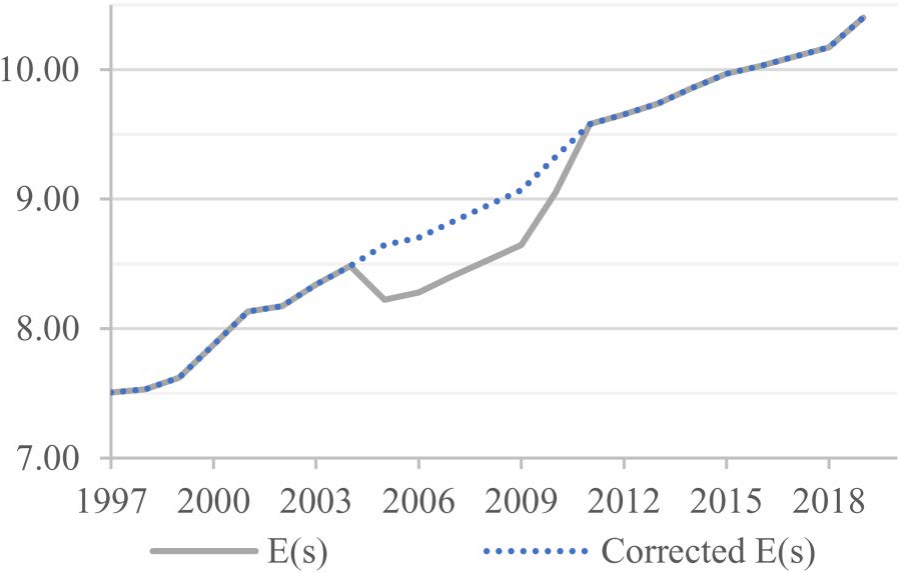
Correction of human capital data. The solid line shows the human capital data for China for each year, the dashed line shows the corrected data.

However, in the calculation of the human capital stock, we observe an anomaly in the data on the education of the employed in China, as shown by the dented section in Fig 1. This anomaly is surprising given China’s sustained economic growth, continuous increase in education investment, and steady improvement in labor quality. The data graph should be relatively smooth, and sudden declines in labor quality should not be present. Therefore, we have made adjustments to the abnormal year data (2005–2010) as follows: We calculate the mean value of E(*s*) differential from 2006-2010 and denote it as AE(*s*). We use this value to adjust the data for 2005, which is equal to the value of 2004 plus the mean AE(*s*). For 2006–2009, we adjust the data by adding the differential term to the value of the previous year. Lastly, we take the mean value of 2009 and 2011 as the data for 2010. With these adjustments, the corrected data exhibit a smooth time dimension and are more consistent with the actual situation.

### Capital stock

We use the perpetual inventory method to calculate the capital stock [30]. This method necessitates the collection of historical data on fixed asset investment, fixed asset price indices, and the determination of the base period capital stock and depreciation rate. The data on annual investments and fixed asset price indices are obtained from the *China Statistical Yearbook*. Price indices reflect the nominal prices for the respective year, which need to be deflated from nominal quantities and then computed into constant-price indices with 1997 as the base year. The depreciation rate is set at 5.26%, as determined in a prior study. The base period capital stock is derived by dividing the total fixed capital formation in 1997 by the depreciation rate.

### GDP and GNI

Both GDP and GNI data are sourced from the annual *China Statistical Yearbook* and need to be adjusted to constant prices for our analysis. Since this paper’s data analysis employs 1997 as the base year, we adjust these figures using price indices published in the *China Statistical Yearbook*, with 1978, the first year of China’s reform and opening up, as the base year, to calculate constant-price indices with 1997 as the base year. Subsequently, we adjust GDP and GNI to constant-price data using the price indices with 1997 as the base year.

### Rates of return for capital and labor factors

We process the data using the following method. Rate of return for labor *w* equals household sector GNI divided by labor force (or human capital stock) and rate of return for capital *r* equals enterprise sector GNI divided by physical capital stock. Data from the *China Statistical Yearbook* for previous years. It is important to note that the GNI data for each sector have been adjusted to 1997 as the base year. Among the data published by the National Bureau of Statistics, the Flow of Funds Accounts is the sole source that directly reports income data for households and corporate sectors. China has been compiling the Flow of Funds Accounts since 1992, and the data is currently updated through 2018.

## Methodology

### Laying the groundwork for indicator construction

General equilibrium theory posits that, under ideal conditions, including a perfectly competitive market, economic agents (consumers and producers) who are perfectly rational, and information that is perfectly symmetrical, the market will spontaneously reach an equilibrium and allocate resources in an optimal manner [31]. When cost is non-existent, the financial system, as a constituent of the market, facilitates the seamless flow of funds from the supplier (savers) to the demander (investors), akin to any other factor of production. This process is guided by the price mechanism (the interest rate), thereby achieving Pareto optimality [32]. It is typically assumed that savings in the household sector are equivalent to net investment in the enterprise sector, with no loss during the conversion of monetary assets into physical capital. Financial intermediaries primarily serve as channels for monetary circulation, and in a scenario with no loss during conversion, financial efficiency is considered to be 100% [8]. In this context, the marginal output of physical capital in the enterprise sector should equal the average return on capital for the entire society. Economic researchers familiar with marginal analysis understand that resource allocation is most efficient under such ideal conditions. However, this no-loss scenario is purely theoretical. In reality, there is a significant disparity in China between the marginal output of physical capital in the corporate sector and the average return on capital for the entire society. Financial efficiency cannot reach 100% and is subject to continuous change.

If financial markets were perfectly competitive, the interest rate would be lower, and correspondingly, the financial sector’s share of marginal capital output would also be lower. However, the Chinese financial sector is composed of a mix of a few large banks, numerous small and medium-sized banks, and other financial service providers, operating within a market structure characterized by oligopoly and monopolistic competition. Consequently, the financial sector has consistently maintained a relatively high share of marginal capital output, which can also be understood as the cost paid by the financial sector to fulfil its resource allocation function.

Next, in order to measure financial sector efficiency, a simple model is constructed to serve as the foundation for the selection of indicators. The efficiency of the financial sector is denoted by *E* and the cost by *C*. In order to comprehend the connotation of cost more concretely, *C* is renamed as the cost of financing *C*_*f*_. According to the general equilibrium theory, there should be a negative correlation between the cost of financing *C*_*f*_ and the efficiency of the financial sector *E*. When *C*_*f*_ = 0, the efficiency of the financial sector reaches its maximum. The efficiency function is represented by


$$\begin{array}{c} E=f\left(C_{f}\right), \end{array}$$ (1)

and *f’(C*_*f*_*) < *0. When *C*_*f*_ = 0, *E* achieves its maximum value, *E*_*max*_.

For example, in a simple model of a lending market, assume that the funds supply function is


$$\begin{array}{c} Q_{s}=a-bP-cC_{f}\left(a,b,c>0\right), \end{array}$$ (2a)

the capital demand function is


$$\begin{array}{c} Q_{d}=d+eP-fC_{f}\left(d,e,f>0\right). \end{array}$$ (2b)

At market equilibrium, $Q_{s}=Q_{d}$, i.e.,


$$\begin{array}{c} a-bP-cC_{f}=d+eP-fC_{f}, \end{array}$$ (3)

further solving for


$$\begin{array}{c} P=\frac{a-d-\left(c-f\right)C_{f}}{b+e}. \end{array}$$ (4)

Then, the volume of financial accommodation is


$$\begin{array}{c} Q=a-b\times \frac{a-d-\left(c-f\right)C_{f}}{b+e}-cC_{f}, \end{array}$$ (5)

the derivation of *C*_*f*_ yields


$$\begin{array}{c} \frac{\partial Q}{\partial C_{f}}<0. \end{array}$$ (6)

It has been demonstrated that an increase in cost *C*_*f*_ results in a decrease in the amount of funds mobilized *Q*, consequently leading to a decline in the efficiency *E* of the financial sector. When *C*_*f*_ =  0 (the ideal case), the volume of funds mobilized is maximized for a given supply and demand function, and the efficiency is at its highest.

According to our method of calculating financial efficiency, obtaining data on financial efficiency requires information on the marginal output of capital and the average return on capital for the entire society. We have primarily undertaken the following two aspects of work.

### Amending the production function

To calculate the marginal output of capital, it is necessary to first determine the production function (or input-output relationship). A significant number of empirical studies on production functions directly employ the Cobb-Douglas functional form and treat total factor productivity (TFP) as an exogenous variable, subsequently testing the structural coefficients of the production function. It is argued that this specification is lacking in rigor. Thus, this paper introduces the following improvements: first, it validates the form of the production function, and second, it adjusts TFP to better control unobservable productivity factors and eliminate endogeneity. Labor and human capital stock data are employed to determine variable and functional forms that better align with reality, eliminating the influence of changes in effective labor input due to endogeneity [14,15].

Firstly, we test the production function instead of directly setting it in Cobb-Douglas form. Because it is difficult to ascertain the specific production characteristics in China (which may lie within its production possibility frontier), we use the term “input-output relationship” to represent the production function for the time being. It is assumed that the input-output relationship of the representative firm conforms to the neoclassical production function, which is given by


$$\begin{array}{c} x=F\left(l,k\right), \end{array}$$ (7)

subjects to


$$\frac{\partial x}{\partial l}\geq 0,\frac{\partial x}{\partial k}\geq 0,\frac{\partial^{2}x}{\partial l^{2}}\leq 0,\frac{\partial^{2}x}{\partial k^{2}}\leq 0,\frac{\partial^{2}x}{\partial l\partial k}\geq 0,$$

where *x* is output (added value), *l* is labor input, *k* is the physical capital stock. Assume that the market price of *x* is *p*(*x*), the market price of labor is *w*, and the average gross market rate of return on physical assets (including depreciation *δk*) is *r*. The firm’s product is in an imperfectly competitive market, and the firm’s supply decision has an effect on the product price *p* and no effect on the factor market price.

The expression for corporate profit π is as follows:


$$\begin{array}{c} \pi =p\cdot x-w\cdot l-r\cdot k, \end{array}$$ (8)

and first-order maximizing condition by


$$\{\begin{array}{c} \frac{\partial \pi }{\partial l}=\left(1+\frac{dp}{dx}\cdot \frac{x}{p}\right)\cdot p\cdot \frac{\partial x}{\partial l}-w=\left(1-\frac{1}{\varepsilon_{x,p}}\right)\cdot p\cdot x_{l}-w=0\\ \frac{\partial \pi }{\partial k}=\left(1+\frac{dp}{dx}\cdot \frac{x}{p}\right)\cdot p\cdot \frac{\partial x}{\partial k}-r=\left(1-\frac{1}{\varepsilon_{x,p}}\right)\cdot p\cdot x_{k}-r=0\\ \varepsilon_{x,p}=-\frac{dx}{dp}\cdot \frac{p}{x},x_{l}=\frac{\partial x}{\partial l},x_{k}=\frac{\partial x}{\partial k} \end{array}.$$

We find the solution characteristic of the marginal rate of technology substitution (MRTS) equal to the economic substitution rate (ESR),


$$\begin{array}{c} MRTS=\frac{x_{l}}{x_{k}}=\frac{w}{r}=ESR. \end{array}$$ (9)

It is used as a basis for determining the form of the production function.

Assume that technological progress obeys Hicks neutrality, the C-D (Cobb-Douglas) and the CES (Constant Elasticity of Substitution) production functions can be represented as follows:


$$\begin{array}{c} x=TFP\cdot l^{\alpha_{l}}\cdot k^{\alpha_{k}}, \end{array}$$ (10a)

$$\begin{array}{c} x=TFP\cdot {\left(\alpha_{l}\cdot l^{\rho }+\alpha_{k}\cdot k^{\rho }\right)}^{\frac{1}{\rho }}. \end{array}$$ (10b)

F.O.C.,


$$\frac{\alpha_{l}}{\alpha_{k}}\cdot \frac{k}{l}=\frac{w}{r},$$

$$\frac{\alpha_{l}}{\alpha_{k}}\cdot {\left(\frac{l}{k}\right)}^{\rho -1}=\frac{w}{r}.$$

Next, we consider human capital *h* as a factor in the input-output relationship. The knowledge and acquired skills possessed by the labor force can be accumulated and upgraded, which increase labor productivity and have characteristics similar to those of physical capital. The channels through which human capital is accumulated by the labor force include both education and “learning by doing”. The corresponding first-order conditions for the optimal allocation of human capital and physical capital are


$$\frac{\alpha_{h}}{\alpha_{k}}\cdot \frac{k}{h}=\frac{w}{r},$$

$$\frac{\alpha_{h}}{\alpha_{k}}\cdot {\left(\frac{h}{k}\right)}^{\rho -1}=\frac{w}{r}.$$

If the relationships between k/l and w/r, as well as k/h and w/r, all exhibit linear relationships with constant coefficients, then it is appropriate to specify the Chinese input-output function in Cobb-Douglas (C-D) form.

Second, we adjust TFP to control for unobservable productivity changes. Since Solow [13] makes seminal contributions to understanding the role of factors other than capital and labor in economic growth, there has been ongoing debate over the methods and accuracy of TFP measurement. Arrow [24] argues that “learning by doing” accumulates in the production of capital goods, leading to both quantitative and qualitative changes in physical capital. This phenomenon can result in fluctuations in TFP. With changing technology and capital quality, many researchers have found that the traditional framework, which constructs functions and metrics based on technological progress and input factors, is endogenous and leads to biased estimates of structural parameters [19,20]. Hence, this paper devotes a substantial portion of its content to examining the optimal form of TFP (see S1 Appendix).

We find that human capital accumulation within the current education system does not contribute significantly to China’s national economic production. Thus, labor quantity data should be employed in computing input factors. By taking a per capita approach, we eliminate labor variables, leaving us with a production function that relies solely on per capita capital as an input factor. This approach greatly facilitates our estimation of structural parameters and the determination of TFP form. Therefore, we develop a two-stage semi-parametric model of China’s national economy input-output,


$$\begin{array}{c} \{\begin{array}{cc} \text{Stage One: } & lngdp=lnTFP^{\circ }+\alpha \cdot lnk+u_{1},\alpha >0\\ \text{Stage Two: } & TFP=\hat{TFP^{\circ }}+\hat{u}=\lambda_{0}+\lambda_{1}\cdot TFP\left(K\right)+v_{1}\\ & lngdp=ln\hat{TFP}+\alpha \cdot lnk+u_{2},\alpha >0 \end{array}. \end{array}$$ (11)

To enhance the accuracy of the estimation results, we adopt a two-stage semi-parametric estimation method that avoids the impact of strong assumptions about data characteristics. The rationale for using a two-stage model is that if the model parameters are estimated directly using the log-transformed one-stage model, the TFP estimation is not optimal after conversion back to the original model when the log residual is not normally distributed. It will ultimately lead to inaccurate estimates of financial efficiency. A semi-parametric model is used to describe the TFP in the first stage, and the parameters *λ*_*0*_ and *λ*_*1*_ are calibrated in the second stage. Semi-parametric estimation is a statistical method that combines the advantages of parametric and non-parametric estimation. In traditional statistical modelling, parametric models are based on assumptions about the overall distribution, with a finite number of parameters describing the relationship between variables. For example, a linear regression model $y=\beta_{0}+\beta_{1}x_{1}+\cdots +\beta_{p}x_{p}+\in$, where $\beta_{i}$ is parameter, ε is error term. It assumes a linear relationship between the variables y and x and that the error term obeys a specific distribution (e.g., normal). Non-parametric models, conversely, demand less rigidity in the overall distribution and variable relationships, and they depend considerably on the data itself to calibrate the model, such as kernel density estimation. Semi-parametric estimation occupies an intermediate position between these two approaches, incorporating components of both parametric and non-parametric methods. Typically, a parametric structure exists to describe the relationship between certain variables; however, no strict parametric assumptions are made about the relationships in other parts, which are estimated using non-parametric methods. Semi-parametric modeling provides greater flexibility than parametric modeling, as it is capable of accommodating situations where the relationship between variables is more complex and cannot be adequately described by parametric forms, such as linear or polynomial models. In cases where the actual data distribution deviates from the assumed distribution in parametric models, the resulting estimates may be biased. The nonparametric component of the semi-parametric model can help mitigate this bias by allowing for a more adaptable representation of the underlying data structure.

In regression, the Jarque-Bera (JB) and Durbin-Watson (DW) tests are used to assess how well the model is fitted. The JB test assesses whether residuals are normally distributed by examining skewness and kurtosis. In the Jarque-Bera test, the closer the skewness is to 0 and the kurtosis is to 3, the more the data approximate a normal distribution. The DW test checks for autocorrelation in regression residuals. A DW statistic around 2 indicates no autocorrelation; values significantly below 2 suggest positive autocorrelation, while values significantly above 2 suggest negative autocorrelation. The test’s results are determined by comparing the DW statistic to critical values.

### Calculating marginal product of capital and average return on capital

In this section, Financial Efficiency is abbreviated as FE, Marginal Product of capital *K* is abbreviated as MP, and Average Return on capital *K* is abbreviated as AR. The formula for Financial Efficiency is as follows:


$$\begin{array}{c} FE_{t}=1/\frac{\left(MP_{t}^{K}-AR_{t}^{K}\right)}{MP_{t}^{K}} \end{array}$$ (12)

We need to derive the marginal product of capital based on the adjusted input-output relationships specific to China, as well as obtain the rate of return on capital based on macroeconomic accounting.

## Results

Following the steps outlined in the Methodology section, this paper first verifies the form of the national economic production function. Based on the identified form of the production function, TFP and the production function are gradually adjusted, ultimately deriving a production function that includes deterministic structural parameters. From this production function, the marginal product of capital (*MP*) is obtained. Using the average return on capital (*AR*) derived from statistical accounting, financial efficiency is then calculated.

A brief overview of the key results is presented. The Cobb-Douglas (C-D) form of the production function is supported by macroeconomic data. Total Factor Productivity (TFP) in the production function is treated endogenously, with its fluctuations found to be associated with capital accumulation, independent of labor force and human capital accumulation. The efficiency of the financial sector is strongly correlated with key economic indicators, such as TFP and GDP growth rates. However, since 2015, a divergence has been observed between changes in financial sector efficiency and the main economic indicators. The details of the results are as follows.

### The form of production function

**Fig 2:**
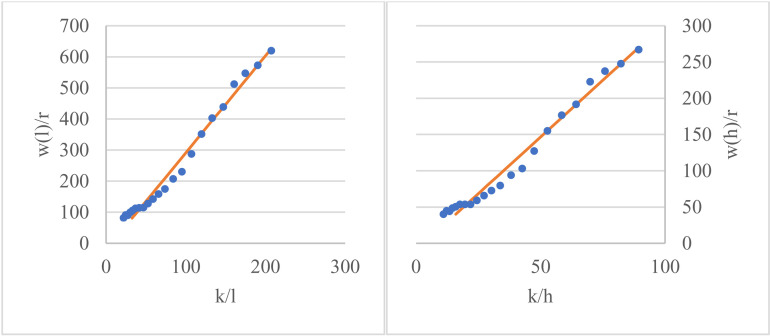
Validation of the functional form of the input-output relationship in China. The scattered data points represent the actual values, while the solid line represents the fitted values after regression. It can be observed that, regardless of whether labor input is measured using labor quantity or human capital stock, the two ratio variables in our optimized solution exhibit a clear linear relationship. This indicates that setting the production function (or input relationship) in Cobb-Douglas form is appropriate.

We examine the relationship between k/l (or k/h) and w/r. Fig 2, taken from 1997-2018, shows that Chinese national economic production basically conforms to the type of production organization described by the C-D function.

### The adjustment of TFP and production function

The residuals from the regression in equation (11) pass both the JB test (skewness is 0.01, kurtosis is 2.20) and DW test (1.69), indicating that the residuals satisfy the tests for goodness of fit to normal distribution with regards to skewness and kurtosis and also pass the test for autocorrelation. This suggests that the model’s specification regarding TFP is reasonable. Using equation (11), we obtain the fitted values for China’s TFP over the years.

**Fig 3:**
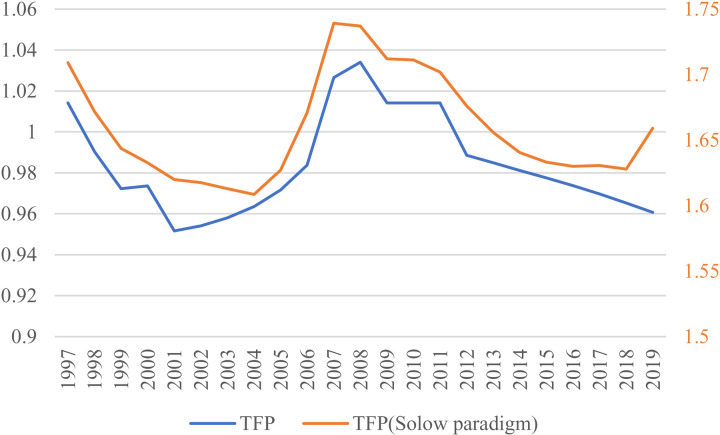
TFP calculated by our method and TFP obtained by Solow’s framework.

Compared to the unadjusted total factor productivity (Solow residual), the two-stage semi-parametric estimation reveals significant differences Fig 3. It indicates that TFP reached its peak in 2008, not in 2007, and that the decline in TFP since 1996 was fundamentally reversed in 2001, not 2004. Moreover, TFP had been consistently declining since 2012, rather than the slowing decline observed before the adjustment. In 2019, TFP was only at 92.9% of its 2008 level. The most crucial and informative distinction in our estimation results is the ongoing downward trend in China’s TFP, rather than a deceleration, and the underlying causes of this trend deserve the attention of policymakers and researchers. These changes suggest that certain biases in prior studies may have been overlooked.

On the left are the axes of TFP computed according to our model, and on the right are the axes of TFP computed in the traditional Solow framework.

The regression results following equation (11) are presented in Table 1. The elasticity coefficient for the capital input is 0.783 (retained to four decimal places, 0.7827), and consequently, the elasticity coefficient for the labor input is 1 − 0.783 = 0.217.

**Table 1.** Regression results for equation (11).

Vars	lnTFPº	lnk	Constant	R^2^	Adj.R2
lngdp	1.0000^***^	0.7827^***^	-0.0000	0.9999	0.9999

*Notes*: *  p <  0.1, ** p <  0.05, *** p <  0.01, the same as below.

Finally, we obtained a national economic production function that aligns with real-world conditions in terms of input statistics, elasticity coefficients, and variable forms：


$$\begin{array}{c} GDP_{t}=TFP\left(K_{t}\right)\cdot L_{t}^{0.217}\cdot K_{t}^{0.783} \end{array}$$ (13)

## Financial efficiency

Based on equation (13), we calculate China’s historical capital marginal output. Utilizing statistical accounting, we derive the capital’s average return. Subsequently, we compute financial efficiency according to equation (12), and the results are presented in Table 2. It’s important to note that, due to the limitations of available data, our calculations are only available up to 2018, as the Chinese National Bureau of Statistics has released financial flow statements only up to that year. Over the sample period, the efficiency of China’s financial sector can be divided into three distinct phases: it steadily increased from 1998, reaching its peak in 2008. Then it began to decline from 2008, hitting a low point in 2015, with financial efficiency in 2015 equivalent to 91.48% of that in 2008. Following this, it started to rise again, with the financial efficiency in 2018 being 93.87% of that in 2008.

**Table 2.** China’s financial efficiency (FE) values, 1997-2018.

Year	FE	Year	FE
1997	1.4121	2008	1.5918
1998	1.3835	2009	1.5568
1999	1.3911	2010	1.5529
2000	1.3911	2011	1.5060
2001	1.4198	2012	1.4784
2002	1.4262	2013	1.4695
2003	1.4796	2014	1.4768
2004	1.5375	2015	1.4562
2005	1.5433	2016	1.4686
2006	1.5545	2017	1.4869
2007	1.5620	2018	1.4942

**Fig 4:**
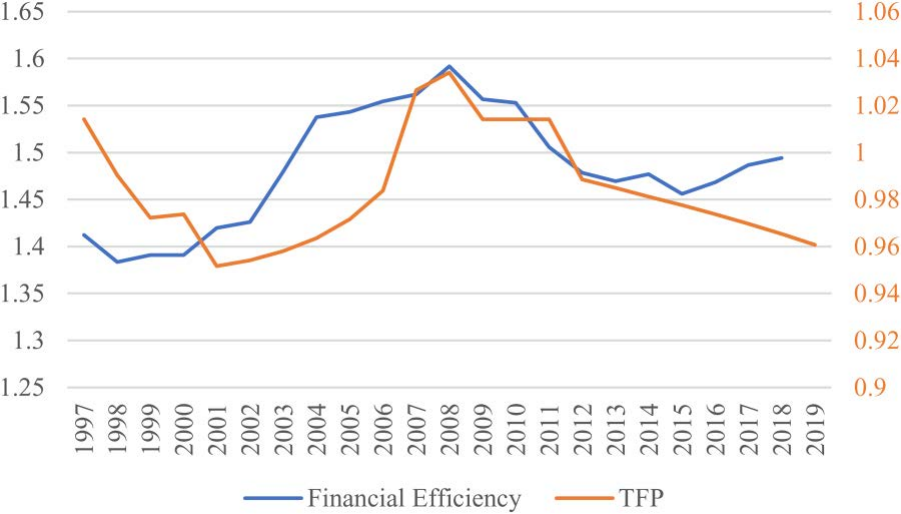
Financial efficiency and TFP in China.

As we utilize TFP and GDP in the calculation of financial efficiency (FE), and because the relationship between FE and productivity as well as national economic output has been a subject of considerable research interest, we choose to compare FE with two indicators, TFP and GDP growth rate, to observe their trends. The trend of FE shows significant similarity with that of TFP. Fig 4 illustrates that changes in FE seem to lead TFP slightly. Initially, TFP declined along with the decrease in FE, then it increased in tandem with the rise in FE, reaching their peak together in 2008. Subsequently, TFP started declining along with the decrease in FE, but in 2015, the two indicators exhibited a divergence in trends, with FE reversing its direction and moving upwards, while TFP continued to decline.

The left vertical coordinate is the financial efficiency value, and the right vertical coordinate is the TFP value.

**Fig 5:**
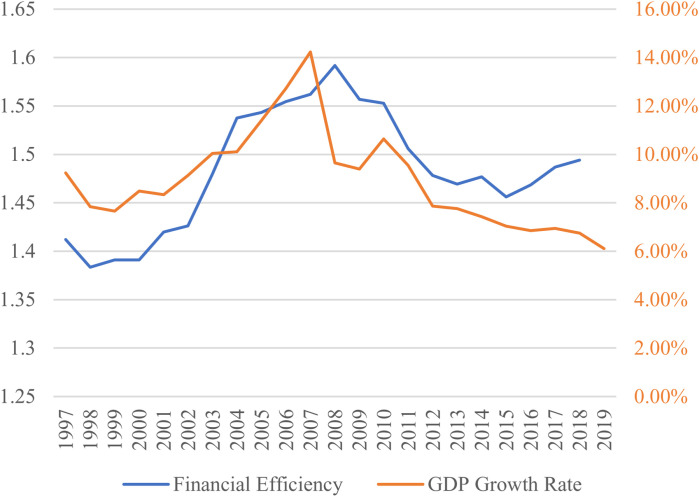
Financial efficiency and GDP growth rate in China.

Next, we compare the trends in financial efficiency with GDP (constant price) growth rate and find a strong correlation between FE and GDP growth rates. Upon observation from Fig 5, it appears that the changes in FE are reasonably synchronized with changes in GDP growth rate, without any evident dominant effect of one on the other. It is worth noting that, similar to the relationship between FE and TFP, there is a divergence in the trends of FE and GDP growth rates in 2015, indicating that the improvement in FE may not have exerted a driving effect on GDP growth rates.

The left vertical coordinate is the financial efficiency value, and the right vertical coordinate is the GDP growth rate.

It is evident that the significant improvement in financial efficiency began in 2001, coinciding with China’s accession to the World Trade Organization (WTO). This suggests that the leading role of financial efficiency in TFP may be attributed to the positive effects of China’s WTO membership, which peaked in 2008. However, starting in 2009, a decline in financial efficiency led to a continuous drop in TFP, attributed to a disconnect between China’s financial system and the real economy. This prompted the Chinese government to undertake substantial reforms of the financial system around 2015, resulting in a rebound in financial efficiency. Nonetheless, TFP continued to decline, possibly due to other factors. The trends in Fig 5 mirror those in Fig 4, showing a distinct two-phase pattern in the relationship between financial efficiency and GDP growth. This indicates that financial efficiency may have had a significant impact on China’s economic growth before 2015. However, the simultaneous occurrence of rising financial efficiency and declining economic growth post-2015 suggests potential issues in the alignment between the financial and economic systems. From the perspective of the production function, the observation that capital is being utilized more efficiently while GDP growth continues to decline indicates significant changes in other economic factors influencing GDP and overall economic growth. This suggests that the decline in growth is not solely attributable to inefficiencies in capital or labor utilization. Considering the optimization and growth of capital and labor inputs, this paper posits that factors such as technological progress, government policies, institutional frameworks, and external shocks may play a pivotal role in driving these changes.

## Concluding remarks

### Main conclusions

We develop a novel approach to measure financial sector efficiency of a nation. From a methodological perspective, this paper establishes the linkage of capital between the financial sector and the real sector, which establishes the basis for the construction of indicator on the measurement of financial efficiency. Based on general equilibrium theory and neoclassical production function, we express financial efficiency as the reciprocal of the ratio of the difference between the marginal output of capital and the average return on capital to the marginal output of capital. Our findings show that over the period from 1997 to 2018 in China, financial efficiency went through an overall pattern of an initial rise, followed by a decline, and then another increase. Financial efficiency seemed to lead TFP positively before 2015, but a divergence between the two emerged afterward. Similarly, there was a substantial correlation between financial efficiency and GDP growth rates before 2015, followed by a divergence beyond that year.

Our approach addresses three shortcomings in evaluating financial efficiency: (1) using a single financial indicator as a proportion of GDP, which lacks theoretical foundation; (2) measuring financial efficiency by selecting financial industry profits or income as a proportion of total turnover, which essentially gauges the operational efficiency of the financial organizations rather than the financial efficiency of the national level; (3) utilizing individually summed data, which may suffer from omissions, errors, and double-counting, making it challenging to reflect the overall performance of the macroeconomic financial sector. Within the framework of general equilibrium theory, this paper quantitatively evaluates financial sector efficiency by the entire financial system using aggregate data. Our approach enriches the research on financial efficiency assessment at the macroeconomic level.

### Implications and limitations

The findings of this paper have implications for both policy and future research: Firstly, regarding policy implications, the trend of declining financial efficiency was reversed in 2015, indicating a significant improvement in the performance of China’s financial system. Based on the theoretical framework and methodology of this paper, the increase in financial efficiency suggests a decrease in the unit cost of supporting national economic development by the financial system. Despite improvements in financial sector efficiency, these advancements have not effectively mitigated or slowed the decline in total factor productivity (TFP) and GDP growth. This highlights the need for policies that establish a strong link between financial sector efficiency and key economic indicators such as TFP and GDP growth. Specifically, policy measures should focus on directing capital accumulation toward the most productive sectors and organizations, fostering competition, and enhancing the efficient allocation of capital to drive TFP growth. One potential policy initiative is the creation of an innovative funding mechanism that integrates government fiscal or monetary instruments with market-based structures. Additionally, policies should aim to reduce frictions within the financial sector, lower barriers to financial access, and incentivize investment in strategic economic activities to ensure that improvements in financial sector efficiency translate into sustained aggregate economic growth. Furthermore, enhancing financial market transparency, promoting competition in capital markets, and adopting accommodative monetary policies are critical directions to reverse the sharp decline in GDP growth and bolster the financial sector’s contribution to economic performance.

Secondly, regarding future research, further investigation is needed to determine whether China’s financial sector efficiency has returned to its 2008 peak level, contingent upon the availability of relevant data. Additionally, the divergence observed since 2015 between the trends of financial efficiency, total factor productivity (TFP), and GDP growth rate requires further examination to understand their interaction mechanisms and potential causality.

This paper also acknowledges certain limitations. It primarily focuses on the design of the financial efficiency methodology and the measurement of financial efficiency over the years, but only provides a preliminary discussion of the causes of changes in financial efficiency and lacks specific empirical evidence and detailed validation.

## Supporting information

S1 Fig The relationship between residuals and growth rates of each variable. The graphical characteristics of the two graphs on the left suggest that physical capital accumulation may be related to residuals.
(DOCX)

S1 Appendix This appendix presents a comprehensive empirical investigation into the application of Cobb-Douglas production functions to analyze the input-output dynamics of China’s national economy. Through rigorous regression analyses (Tables A.1–A.6), the study explores the interplay of Gross Domestic Product (GDP), physical capital (K), human capital (H), and labor (L), with a focus on refining specifications of Total Factor Productivity (TFP). The analysis employs methodological innovations, including structural break adjustments (e.g., 1999–2008 and 2012–2019 periods) and episodic event controls (e.g., financial crises, policy shifts), to enhance model robustness and accuracy. Results reveal nuanced insights: while physical capital consistently demonstrates significant output elasticity, human capital exhibits context-dependent effects.
(DOCX)

## References

[1] Wang Z, Zhang C, Wu R, Sha L. From ethics to efficiency: Understanding the interconnected dynamics of ESG performance, financial efficiency, and cash holdings in China. Finance Research Letters. 2024;64:105419. doi: 10.1016/j.frl.2024.105419

[2] Yuan S, Wu Z, Liu L. The effects of financial openness and financial efficiency on Chinese macroeconomic volatilities. N Am J Econ Financ. 2022;63:101819. doi: 10.1016/j.najef.2022.101819

[3] Levine R. Financial development and economic growth: views and agenda. J Econ Literature. 1997;35(2):688–726.

[4] Beck T, Levine R, Loayza N. Finance and the sources of growth. J Financ Econ. 2000;58(1–2):261–300. doi: 10.1016/s0304-405x(00)00072-6

[5] Hu M, Zhang J, Chao C. Regional financial efficiency and its non-linear effects on economic growth in China. Int Rev Econ Financ. 2019;59:193–206. doi: 10.1016/j.iref.2018.08.019

[6] Guillaumont Jeanneney S, Hua P, Liang Z. Financial development, economic efficiency, and productivity growth: evidence from China. Dev Econ. 2006;44(1):27–52. doi: 10.1111/j.1746-1049.2006.00002.x

[7] Bazot G. Financial consumption and the cost of finance: measuring financial efficiency in Europe (1950–2007). J Eur Econ Assoc. 2017. doi: 10.1093/jeea/jvx008

[8] Mishkin F. The economics of money, banking, and financial markets: Pearson education. 2007.

[9] Collins N, Gottwald J-C. Market creation by leninist means: the regulation of financial services in the People’s Republic of China. Asian Stud Rev. 2014;38(4):620–38. doi: 10.1080/10357823.2014.964173

[10] Dai Z, Guo L. Market competition and corporate performance: empirical evidence from China listed banks with financial monopoly aspect. Appl Econ. 2020;52(44):4822–33. doi: 10.1080/00036846.2020.1745749

[11] Ackerberg D, Chen X, Hahn J, Liao Z. Asymptotic efficiency of semiparametric two-step GMM. Rev Econ Stud. 2014;81(3):919–43. doi: 10.1093/restud/rdu011

[12] Kim K il, Petrin A, Song S. Estimating production functions with control functions when capital is measured with error. J Econometrics. 2016;190(2):267–79. doi: 10.1016/j.jeconom.2015.06.016

[13] Solow RM. Technical change and the aggregate production function. Rev Econ Stat. 1957:312-20.

[14] Young A. The tyranny of numbers: confronting the statistical realities of the East Asian growth experience. Q J Econ. 1995;110(3):641–80. doi: 10.2307/2946695

[15] Olley GS, Pakes A. The dynamics of productivity in the telecommunications equipment industry. Econometrica. 1996;64(6):1263. doi: 10.2307/2171831

[16] Hsieh CT. What explains the industrial revolution in East Asia? evidence from the factor markets. Am Econ Rev. 2002;92(3):502–26. doi: 10.1257/00028280260136372

[17] Levinsohn J, Petrin A. Estimating production functions using inputs to control for unobservables. Rev Econ Studies. 2003;70(2):317–41. doi: 10.1111/1467-937x.00246

[18] Van Biesebroeck J. Productivity dynamics with technology choice: an application to automobile assembly. Rev Econ Studies. 2003;70(1):167–98. doi: 10.1111/1467-937x.00241

[19] Ackerberg DA, Caves K, Frazer G. Identification properties of recent production function estimators. Econometrica. 2015;83(6):2411–51. doi: 10.3982/ecta13408

[20] Hu Y, Huang G, Sasaki Y. Estimating production functions with robustness against errors in the proxy variables. J Econ. 2020;215(2):375–98. doi: 10.1016/j.jeconom.2019.05.024

[21] Zhang Q, Xu Z, Feng T, Jiao J. A dynamic stochastic frontier model to evaluate regional financial efficiency: evidence from Chinese county-level panel data. Eur J Oper Res. 2015;241(3):907–16. doi: 10.1016/j.ejor.2014.09.021

[22] Becker GS. Investment in human capital: a theoretical analysis. J Polit Econ. 1962;70(5):9–49.

[23] Becker GS. Human capital: a theoretical and empirical analysis, with special reference to education. 2009.

[24] Arrow KJ. The Economic Implications of Learning by Doing. 1962.

[25] Angrist JD, Krueger AB. Does compulsory school attendance affect schooling and earnings? Q J Econ. 1991;106(4):979–1014. doi: 10.2307/2937954

[26] Angrist J, Krueger AB. Estimating the payoff to schooling using the Vietnam-era draft lottery. Mass., USA: National Bureau of Economic Research Cambridge; 1992.

[27] Acemoglu D, Angrist J. How large are human-capital externalities? evidence from compulsory schooling laws. NBER Macroeconomics Annual. 2000;15:9–59. doi: 10.1086/654403

[28] Harris DN. Diminishing marginal returns and the production of education: an international analysis. Education Economics. 2007;15(1):31–53. doi: 10.1080/09645290601133894

[29] Carneiro P, Heckman JJ, Vytlacil E. Estimating marginal returns to education. Am Econ Rev. 2011;101(6):2754–81. doi: 10.1257/aer.101.6.2754 25110355 PMC4126808

[30] Berlemann M, Wesselhöft JE. Estimating aggregate capital stocks using the perpetual inventory method. Review of Economics. 2014;65(1):1–34. doi: 10.1515/roe-2014-0102

[31] Arrow KJ, Debreu G. ‘Existence of an equilibrium for a competitive economy’. Foundations of Price Theory. 2024;5:289–316. doi: 10.4324/9781003547990-14

[32] Burk A. A reformulation of certain aspects of welfare economics. Q J Econ. 1938;52(2):310–34.

