# Supplementary material for: Measuring financial sector efficiency in China: A study based on an enhanced neoclassical production function
Source: PLoS One. 2025 Apr 10;20(4):e0319480. doi: 10.1371/journal.pone.0319480 (PMC11984730; doi:10.1371/journal.pone.0319480)
Supplement: S1 Fig — The graphical characteristics of the two graphs on the left suggest that physical capital accumulation may be related to residuals. (DOCX) [file pone.0319480.s001.docx]

**S1 Figure.** The relationship between residuals and growth rates of each variable.

The graphical characteristics of the two graphs on the left suggest that physical capital accumulation may be related to residuals.
