# Supplementary material for: Measuring financial sector efficiency in China: A study based on an enhanced neoclassical production function
Source: PLoS One. 2025 Apr 10;20(4):e0319480. doi: 10.1371/journal.pone.0319480 (PMC11984730; doi:10.1371/journal.pone.0319480)
Supplement: S1 Appendix — Through rigorous regression analyses (Tables A.1–A.6), the study explores the interplay of Gross Domestic Product (GDP), physical capital (K), human capital (H), and labor (L), with a focus on refining specifications of Total Factor Productivity (TFP). The analysis employs methodological innovations, including structural break adjustments (e.g., 1999–2008 and 2012–2019 periods) and episodic event controls (e.g., financial crises, policy shifts), to enhance model robustness and accuracy. Results reveal nuanced insights: while physical capital consistently demonstrates significant output elasticity, human capital exhibits context-dependent effects. (DOCX) [file pone.0319480.s002.docx]

# Appendix A

After validating the C-D function form, the input-output relationship of China’s national economy is set as

$$\begin{aligned} GDP=TFP\left( H,K \right)\cdot L^{\alpha_{l}}\cdot K^{\alpha_{k}},\alpha_{l},\alpha_{k}>0,\#\left（ A.1a \right） \end{aligned}$$

$$\begin{aligned} GDP=TFP\left( K \right)\cdot H^{\alpha_{h}}\cdot K^{\alpha_{k}},\alpha_{h},\alpha_{k}>0.\#\left（ A.1b \right） \end{aligned}$$

In equation (A.1a), *TFP (H, K)* varies with the accumulation of human capital *H* and physical capital *K*, reflecting the effects of technological progress derived from "learning by education" and "learning by investment". *TFP(K)* in equation (A.1b) depends only on *K*, reflecting the effect of technological progress from "learning by investment".

We ignore the form of TFP first. The equation of empirical validation of TFP is

$$\begin{aligned} lnGDP=lnTFP+\alpha_{l}\cdot ln L+\alpha_{k}\cdot ln K+u.\#\left（ A.2a \right） \end{aligned}$$

Table A.1 shows that *lnTFP* is not significant and may be a variable. The output elasticity $\alpha_{l}$ of labor force *L* is insignificant, possibly because it is not a constant or there is a cointegration between *lnL* and *lnK*. Using a combination of human capital *H* and physical capital *K*, there is not substantially different from equation (A.2b). We speculate that there is no significant output effect of human capital accumulation.

**Table A.1** Regression results for equation (A.2a)

| Variable | lnL | lnK | Constant | R^2^ | Adj.R2 |
| --- | --- | --- | --- | --- | --- |
| lnGDP | 0.4239 | 0.7744*** | -1.2926 | 0.9984 | 0.9982 |

*Notes*: The result is horizontal output rather than a regular vertical one. Dependent variable is *lnGDP* and independent variables are *lnL* and *lnK.* Where *GDP* is gross domestic product, *TFP* is total factor productivity of national economy production, *L* is total employment, and *H* and *K* are the total stock of human capital and physical capital in the productive sectors of the national economy, respectively. There are 24 years of observations (1996-2019).

Using a combination of human capital *H* and physical capital *K*, the equation of empirical validation of TFP is

$$\begin{aligned} lnGDP=ln TFP+\alpha_{h}\cdot ln H+\alpha_{k}\cdot ln K+u.\#\left（ A.2b \right） \end{aligned}$$

**Table A.2** Regression results for equation (A.2b)

| Vars | lnH | lnK | Constant | R^2^ | Adj.R2 |
| --- | --- | --- | --- | --- | --- |
| lnGDP | 0.2483 | 0.7662*** | -0.2357 | 0.9984 | 0.9982 |

We find that equation (A.1b) exhibits greater consistency with the available data. As both (equation A.2a, A.2b) of the above regression models have positive residual series correlation, they need to be further analyzed. Comparing the residual series with the growth rates of physical capital stock *K*, employment size *L* and human capital stock *H*, *g_K_*, *g_L_* and *g_H_* S1, we speculate that physical capital accumulation may influence the residuals.

**S1 Figure.** The relationship between residuals and growth rates of each variable.

The graphical characteristics of the two graphs on the left suggest that physical capital accumulation may be related to residuals.

In essence, the residual *u* is a non-random variable that contains a component of TFP that may be related to the accumulation of physical capital *k* and requires endogenous treatment. Therefore, let *TFP* = *TFP(K)*, whose value is the sum of the intercept and residual term from equation A.2b,

$$\begin{aligned} \hat{TFP}\left( K \right)=e^{\text{intercet }+\hat{u}_{H}}.\#\left（ A.3 \right） \end{aligned}$$

From the estimation of Equation A.2b, it is clear that the value of TFP in Equation A.3 is biased. We find that using 2008 as the boundary, the *u_h_* rises with the accumulation of physical capital before and falls with the accumulation of physical capital after.^[[1]](#footnote-1)^ After several attempts, equation (A.3) is described endogenously as follows:

$$\begin{aligned} TFP \left( K_{t} \right)=\text{ intercept }+\beta_{1}\cdot D_{99-08}\cdot e^{\frac{K_{t}}{K_{97}}-1}+\beta_{2}\cdot D_{99-08}+\beta_{3}\cdot D_{12-19}\cdot\left( \frac{K_{t}}{K_{97}}-1 \right)+v_{t}.\#\left（ A.4 \right） \end{aligned}$$

*D_99-08_* indicates that the variable takes a value of 1 in 1999-2008 and 0 in other years because of time series characteristics of *u_h_*, and so does *D_12-19_*, which indicates that the variable takes a value of 1 in 2012-2019, and 0 in other years. We add (*K_t_/K_97_-1*) to the equation to deal with endogeneity.

**Table A.3** Regression results for equation (A.4)

| Variable | D_99-08_e^K^ | D_99-08_ | D_12-19_K | Constant | R^2^ | Adj.R2 |
| --- | --- | --- | --- | --- | --- | --- |
| TFP(K) | 0.0052*** | -0.0498*** | -0.0037*** | 0.8109*** | 0.8249 | 0.7972 |

*Notes*: The dependent variable is *TFP(K).* For simplicity, we use *D_99-08_e^K^* and *D_12-19_K* to substitute $D_{99-08}\cdot e^{\frac{K_{t}}{K_{97}}-1}$and $D_{12-19}\cdot\left( \frac{K_{t}}{K_{97}}-1 \right)$, respectively.

Table A.3 shows that the regressed indicators are all satisfactory, except for the goodness of fit. It derives from that the equation (A.4) may ignore episodic shocks, such as the Asian financial crisis, producers' favorable expectations for China's WTO accession in 1999/2000, and the incentives for capacity utilization from the heating up of the real estate sector in 2007. The model is adjusted as follows:

$$\begin{aligned} TFP \left( K_{t} \right)=\text{ intercept }+\beta_{1}\cdot D_{99-08}\cdot e^{\frac{K_{t}}{K_{97}}-1}+\beta_{2}\cdot D_{99-08}+\beta_{3}\cdot D_{12-19}\cdot\left( \frac{K_{t}}{K_{97}}-1 \right)+impact \\ +v_{t},\#\left（ A.5 \right） \end{aligned}$$

where *impact* refers to episodic event shocks and takes a value of -1 in 1998, 1 in 1999,2000,2007 and 0 in the remaining years.

Table A.4 indicates that the fitting degree of the modified model and the unbiasedness of the model are significantly improved. At the same time, the corrections to the model do not affect the characteristics of the random distribution of the residual terms.

**Table A.4** Regression results for equation (A.5)

| Variable | D_99-08_e^K^ | D_99-08_ | D_12-19_K | impact | Constant | R^2^ | Adj.R2 |
| --- | --- | --- | --- | --- | --- | --- | --- |
| TFP(K) | 0.0054*** | -0.0607*** | -0.0041*** | 0.0196*** | 0.8145*** | 0.9544 | 0.9442 |

In order to exclude the possible interference caused by multicollinearity, equation (A.2b) is transformed as follows

$$\begin{aligned} \ln Y=\ln TFP+\alpha_{h}\cdot\ln H+\alpha_{k}\cdot\ln K\Rightarrow g_{Y}=g_{TFP}+\alpha_{h}\cdot g_{H}+\alpha_{k}\cdot g_{K}.\#\left（ A.6 \right） \end{aligned}$$

In equation (A.6), there is essentially no correlation between the growth rate of human capital, *g_H_*, and the growth rate of physical capital, *g_K_*, while the output elasticity *α_h_* of human capital *H* becomes negative, as shown in table A.5. Therefore, it could be concluded that there is indeed a surplus of labor and human capital in China’s national economic production, and no marginal output effect is produced by increasing employment or human capital accumulation.

**Table A.5** Regression results for equation (A.6)

| Vars | gTFP | gH | gK | Constant | R^2^ | Adj.R2 |
| --- | --- | --- | --- | --- | --- | --- |
| gGDP | 0.9027*** | -0.0291 | 0.7422*** | 0.5143 | 0.8681 | 0.8449 |

Consequently, none of the above models is suitable for describing the input-output relationship of China’s national economy. Thus, the results of TFP estimated by equation (A.5) are not credible.

Therefore, to still be able to estimate the true input-output relationship, the equation in aggregate form needs to be transformed by replacing *GDP* and *K* with *GDP* per capita, *gdp*, and the stock of physical capital per employed person, *k*,

$$\begin{aligned} gdp=TFP\cdot\left( \frac{L}{L} \right)^{1-\alpha}\cdot\left( \frac{K}{L} \right)^{\alpha}=TFP\cdot k^{\alpha},\alpha>0,\#\left( A.7a \right) \end{aligned}$$

$$\begin{aligned} gdp=TFP\cdot\left( \frac{H}{L} \right)^{1-\alpha}\cdot\left( \frac{K}{L} \right)^{\alpha}=TFP\cdot h^{1-\alpha}\cdot k^{\alpha},h=\ln E\left( s \right),\alpha>0.\#\left( A.7b \right) \end{aligned}$$

Take the logarithm of equation (A.7a) and (A.7b) and then regress. The output elasticity of human capital of equation (A.7b) is significantly negative, that is, human capital accumulation is negatively related to national economic output, which is normally not consistent with common sense. This suggests that it is not reasonable to use the stock of human capital as an input to the factor of labor.

**Table A.6** Regression results for equation (A.7a) and (A.7b)

| Vars | | lnk | | Constant | | R^2^ | | Adj.R2 | |
| --- | --- | --- | --- | --- | --- | --- | --- | --- | --- |
| lngdp | | 0.7792*** | | 0.0005 | | 0.9982 | | 0.9982 | |
| Vars | lnk | | lnh | | Constant | | R^2^ | | Adj.R2 |
| lngdp | 0.8719*** | | -1.3791*** | | 0.6732*** | | 0.9987 | | 0.9986 |

1. The trend of *u_l_* is the same as *u_h_*. [↑](#footnote-ref-1)
